# CLEFMA Activates the Extrinsic and Intrinsic Apoptotic Processes through JNK1/2 and p38 Pathways in Human Osteosarcoma Cells

**DOI:** 10.3390/molecules24183280

**Published:** 2019-09-09

**Authors:** Jia-Sin Yang, Renn-Chia Lin, Yi-Hsien Hsieh, Heng-Hsiung Wu, Geng-Chung Li, Ya-Chiu Lin, Shun-Fa Yang, Ko-Hsiu Lu

**Affiliations:** 1 Department of Medical Research, Chung Shan Medical University Hospital, Taichung 402, Taiwan; 2 Institute of Medicine, Chung Shan Medical University, Taichung 402, Taiwan (R.-C.L.) (Y.-C.L.); 3 Department of Orthopedics, Chung Shan Medical University Hospital, Taichung 402, Taiwan; 4 School of Medicine, Chung Shan Medical University, Taichung 402, Taiwan; 5 Division of Hyperbaric Oxygen Therapy and Wound Medicine, Chung Shan Medical University Hospital, Taichung 402, Taiwan; 6 Institute of Biochemistry, Microbiology and Immunology, Chung Shan Medical University, Taichung 402, Taiwan (Y.-H.H.) (G.-C.L.); 7 Graduate Institute of Biomedical Science, China Medical University, Taichung 404, Taiwan; 8 Research Center of Tumor Medical Science, China Medical University, Taichung 404, Taiwan; 9 Center for Molecular Medicine, China Medical University Hospital, Taichung 404, Taiwan

**Keywords:** apoptosis, CLEFMA, JNK, osteosarcoma, p38

## Abstract

Due to the poor prognosis of metastatic osteosarcoma, chemotherapy is usually employed in the adjuvant situation to improve the prognosis and the chances of long-term survival. 4-[3,5-Bis(2-chlorobenzylidene)-4-oxo-piperidine-1-yl]-4-oxo-2-butenoic acid (CLEFMA) is a synthetic analog of curcumin and possesses anti-inflammatory and anticancer properties. To further obtain information regarding the apoptotic pathway induced by CLEFMA in osteosarcoma cells, microculture tetrazolium assay, annexin V-FITC/PI apoptosis staining assay, human apoptosis array, and Western blotting were employed. CLEFMA dose-dependently decreased the cell viabilities of human osteosarcoma U2OS and HOS cells and significantly induced apoptosis in human osteosarcoma cells. In addition to the effector caspase 3, CLEFMA significantly activated both extrinsic caspase 8 and intrinsic caspase 9 initiators. Moreover, CLEFMA increased the phosphorylation of extracellular signal-regulated protein kinases (ERK)1/2, c-Jun N-terminal kinases (JNK)1/2 and p38. Using inhibitors of JNK (JNK-in-8) and p38 (SB203580), CLEFMA’s increases of cleaved caspases 3, 8, and 9 could be expectedly suppressed, but they could not be affected by co-treatment with the ERK inhibitor (U0126). Conclusively, CLEFMA activates both extrinsic and intrinsic apoptotic pathways in human osteosarcoma cells through JNK and p38 signaling. These findings contribute to a better understanding of the mechanisms responsible for CLEFMA’s apoptotic effects on human osteosarcoma cells.

## 1. Introduction

Osteosarcoma, the most common histological form of primary bone cancer, is most prevalent in teenagers and young adults [1,2]. Surgical *en bloc* resection of the cancer to achieve a complete radical excision has been the treatment of choice for osteosarcoma [2], but its prognosis is poor because of its highly metastatic potential. To decrease its high treatment failure and mortality rates, the combination of surgery and chemotherapy for osteosarcoma has increased long-term survival chances to approximately 68% through limb-sparing surgeries based on radiological staging, surgical techniques, and new chemotherapy protocols [2,3]. Nevertheless, potent metastatic lung diseases are still responsible for one of the most lethal pediatric malignancies to date. Because of this, novel agents that target particular intracellular signaling pathways related to the distinctive properties of osteosarcoma cells need to be developed.

Apoptosis, or programmed cell death, a key regulator of physiological growth control and regulation of tissue homeostasis, is characterized by typical morphological and biochemical hallmarks, including cell shrinkage, nuclear DNA fragmentation and membrane blebbing [4]. Multiple stress-inducible molecules, such as mitogen-activated protein kinase (MAPK)/extracellular signal-regulated protein kinase (ERK), c-Jun N-terminal kinase (JNK), and nuclear factor kappa B (NF-κB), have been implied in transmitting the apoptotic pathway [5,6]. To undergo apoptosis, the activation of important initiator and effector caspases would be initiated through the activation of the extrinsic (receptor) pathway or the stimulation of the intrinsic (mitochondria) pathway [7,8,9]. Currently, most anticancer strategies in clinical oncology focus on triggering apoptosis in cancer cells. On the contrary, failure to undergo apoptosis may result in treatment resistance. Thereby, understanding the molecular events that regulate apoptosis in response to chemotherapy provides novel opportunities to develop molecular-targeted therapy through the intrinsic and/or extrinsic pathways for osteosarcoma, which is very difficult to cure.

Curcumin (diferuloylmethane), a bright yellow chemical produced by Curcuma longa plants, has been shown to exhibit antioxidant, anti-inflammatory, antibacterial, antiviral, antifungal, and anticancer activities through the modulation of multiple cell signaling pathways [10]. The potent cytotoxic activity of curcumin on osteosarcoma cells has been reported to be mediated by the induction of multiple apoptotic processes [11,12,13,14,15]. However, even though curcumin is safe at high doses (12 g/day) for humans, many reasons, such as its poor absorption, rapid metabolism, and rapid systemic elimination, contribute to the low plasma and tissue levels of curcumin [16]. To improve the poor bioavailability of curcumin, numerous approaches have been undertaken, including the use of adjuvants and structural analogues of curcumin (e.g., EF24 [3,5-bis(2-fluorobenzylidene) piperidin-4-one]).

4-[3,5-Bis(2-chlorobenzylidene)-4-oxo-piperidine-1-yl]-4-oxo-2-butenoic acid (CLEFMA) is a synthetic analog of EF 24 and possesses anti-inflammatory and anticancer properties [17,18]. Using a reverse-phase high-performance liquid chromatography (HPLC) method to analyze the stability of the new drug, CLEFMA has been validated as a potential active anticancer drug-product [19]. In fact, various signaling pathways involved in diverse antitumor properties all depend on different specific tumor types and cell lines. Despite the absence of apoptosis, the curcuminoid CLEFMA has an anti-proliferative activity to induce autophagic cell death via oxidative stress in human lung adenocarcinoma H441 cells, offering an alternative mode of cell death in apoptosis-resistant cancers [17]. Moreover, CLEFMA-induced cell death and tumor growth suppression has been reported to be associated with the cleavage of caspases 3/9 and NF-κB-regulated anti-inflammatory and anti-metastatic effects [20]. As a potent diphenyldihaloketone analogue, CLEFMA has been developed over the past years as an anticancer agent [17]; nonetheless, the effect of CLEFMA on human osteosarcoma cell death remains unclear. Thus, we investigated whether CLEFMA affects the apoptosis of osteosarcoma and attempted to define its underlying mechanisms.

## 2. Results

### 2.1. Cytotoxicity of CLEFMA in Osteosarcoma U2OS and HOS Cells

**Figure 1:**
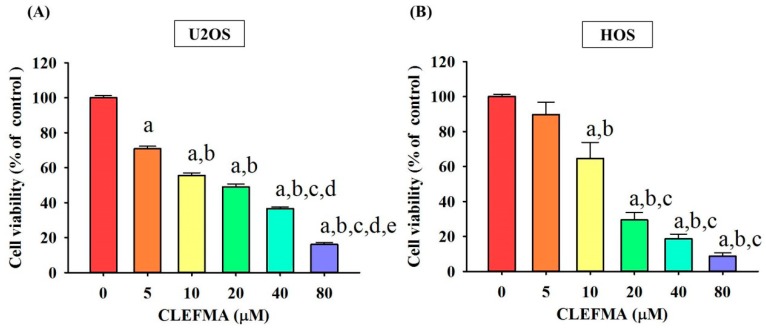
Effects of 4-[3,5-Bis(2-chlorobenzylidene)-4-oxo-piperidine-1-yl]-4-oxo-2-butenoic acid (CLEFMA) on the cell viability of U2OS and HOS cells. Using an [3-(4,5-dimethylthiazol-2-yl)-2,5-diphenyltetrazolium bromide] (MTT) assay, the viability of U2OS and HOS cells treated with CLEFMA (5, 10, 20, 40 and 80 μM) for 24 h was detected, and the effects are illustrated after quantitative analysis. Results are shown as mean ± S.D. (**A**) *n* ≥ 4. ANOVA analysis with Scheffe’s posteriori comparison was used. F = 386.619, *p* < 0.001. (**B**) *n* ≥ 4. ANOVA analysis with Turkey’s posteriori comparison was used. F = 53.288, *p* < 0.001. a: Significantly different, *p* < 0.05, when compared to control. b: Significantly different, *p* <0.05, when compared to 5 μM. c: Significantly different, *p* < 0.05, when compared to 10 μM. d: Significantly different, *p* < 0.05, when compared to 20 μM. e: Significantly different, *p* < 0.05, when compared to 40 μM.

To assess the cytotoxicity of CLEFMA on osteosarcoma U2OS and HOS cells, the [3-(4,5-dimethylthiazol-2-yl)-2,5-diphenyltetrazolium bromide] (MTT) assay was utilized. After 24 h of treatment, the viabilities of U2OS and HOS cells in the presence of concentrations of 5, 10, 20, 40 and 80 μM of CLEFMA were significantly different to that of the controls (0 μM) (Figure 1A,B), and both of the relationships were dose-dependent (*p* < 0.001 and *p* < 0.001). Moreover, a 24 h treatment with 20 μM of CLEFMA showed about a 50% reduction, while a 24 h treatment with 80 μM of CLEFMA decreased the cell viability of U2OS cells by about 90%. In HOS cells, there were reductions of about 70% in 20 μM and about 90% in 80 μM of CLEFMA.

### 2.2. CLEFMA Induces the Apoptosis of U2OS and HOS Cells

**Figure 2:**
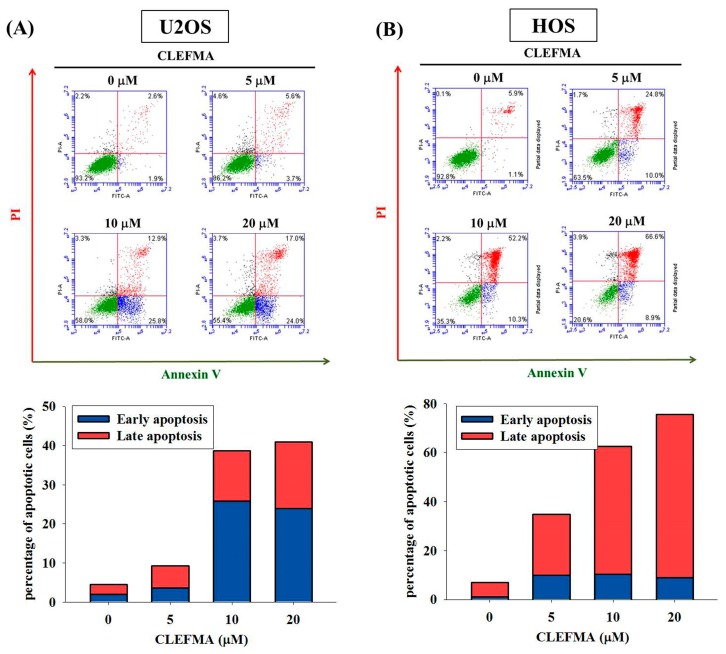
Effects of CLEFMA on the apoptosis of U2OS and HOS cells. (**A**) U2OS and (**B**) HOS cells were treated with CLEFMA (5, 10 and 20 μM) for 24 h and then subjected to flow cytometry after annexin V-FITC/PI staining. Cells that were considered viable were FITC annexin V and PI negative, cells that were in early apoptosis were FITC annexin V positive and PI negative, and cells that were in late apoptosis or already dead were both FITC annexin V and PI positive. Thus, the quantitative analysis of early apoptosis and late apoptosis was summarized to differentiate apoptosis from necrosis.

To further examine the mechanism of CLEFMA inhibition of osteosarcoma cell proliferation, the annexin V-FITC/PI apoptosis assay was performed to test the viability of U2OS and HOS cells after a treatment of 5, 10, and 20 μM of CLEFMA for 24 h. The results revealed that the percentage of apoptotic cells was significantly increased in a dose-dependent manner (Figure 2A,B). These findings suggest that CLEFMA induced the apoptosis of osteosarcoma cells.

### 2.3. CLEFMA Increases the Expression of Cleaved Caspase 3 in U2OS Cells

**Figure 3:**
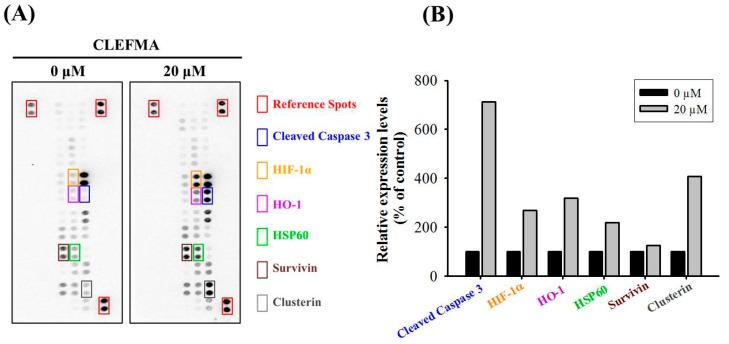
Effects of CLEFMA on the human apoptosis array in U2OS cells. (**A**) After treatment with 20 μM CLEFMA for 24 h in U2OS cells, the human apoptosis array, with 35 apoptosis-related proteins included, was employed as described in the Materials and Methods. (**B**) The five increased proteins were subjected to quantitative analysis.

To identify the underlying mechanism of apoptosis induced by CLEFMA in U2OS cells, we first employed the human apoptosis array to determine apoptosis-related proteins in U2OS cells. Consequently, obvious increases in the expression of cleaved caspase 3, HIF-1α, HO-1, HSP60, survivin and clusterin in U2OS cells were observed after treatment with 20 μM CLEFMA for 24 h. (Figure 3) Among them, the protein that increased the most in quantity was cleaved caspase 3, which was seven-fold that of the original, suggesting that the effector caspase 3 is responsible for the actual dismantling of the U2OS cell.

### 2.4. CLEFMA Triggers Activation of the Caspase Cascade in U2OS Cells

**Figure 4:**
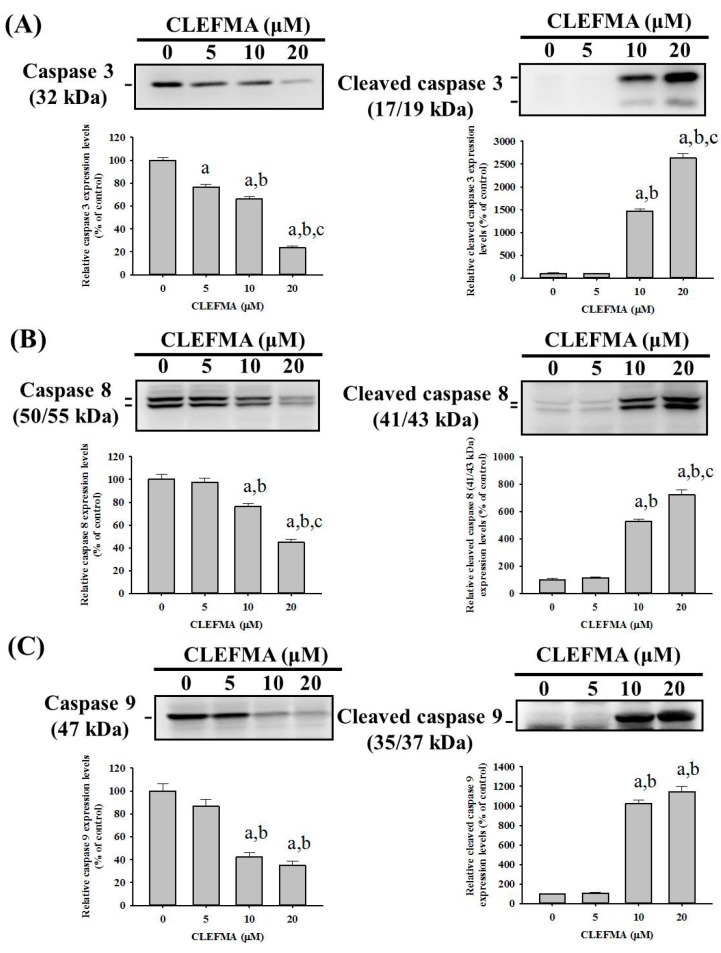
Effects of CLEFMA on the activation of caspases 3, 8 and 9 in U2OS cells. Western blot analysis for caspases 3, 8 and 9 and their active forms after various concentrations (5, 10 and 20 μM) of CLEFMA treatment for 24 h in U2OS cells were measured as described in the Materials and Methods. Subsequently, (**A**) caspase 3 and cleaved caspase 3, (**B**) caspase 8 and cleaved caspase 8, and (**C**) caspase 9 and cleaved caspase 9 were subjected to quantitative analysis. Results are shown as mean ± S.D.; n = 3. ANOVA analysis with Turkey’s posteriori comparison was used. Caspase 3: F = 196.205, *p* < 0.001; cleaved caspase 3: F = 478.594, *p* < 0.001. Caspase 8: F = 51.604, *p* < 0.001; cleaved caspase 8: F = 205.373, *p* < 0.001. Caspase 9: F = 37.754, *p* < 0.001; cleaved caspase 9: F = 294.964, *p* < 0.001. a: Significantly different, *p* < 0.05, when compared to control. B: Significantly different, *p* < 0.05, when compared to 5 μM. c: Significantly different, *p* < 0.05, when compared to 10 μM.

To investigate the effect of CLEFMA on the caspase cascade in the apoptotic signaling pathway, the effector caspase 3 and its upstream initiators, caspases 8 and 9, as well as their cleaved forms were determined with Western blotting. After treatment with different concentrations of CLEFMA in U2OS cells for 24 h, the higher concentrations of CLEFMA corresponded to higher expressions of the cleaved forms of caspases 3, 8, and 9, in a dose-dependent manner (*p* < 0.001, *p* < 0.001 and *p* < 0.001, respectively), combined with the lesser expressions of caspases 3, 8, and 9, dose-dependently (*p* < 0.001, *p* < 0.001 and *p* < 0.001, respectively). (Figure 4A–C) Thus, we found that CLEFMA induces U2OS cell apoptosis by activating both extrinsic caspase 8- and intrinsic caspase 9-mediated pathways and their downstream effector caspase 3.

### 2.5. CLEFMA Activates Extrinsic and Intrinsic Apoptotic Processes via JNK and p38 Pathways in U2OS Cells

**Figure 5:**
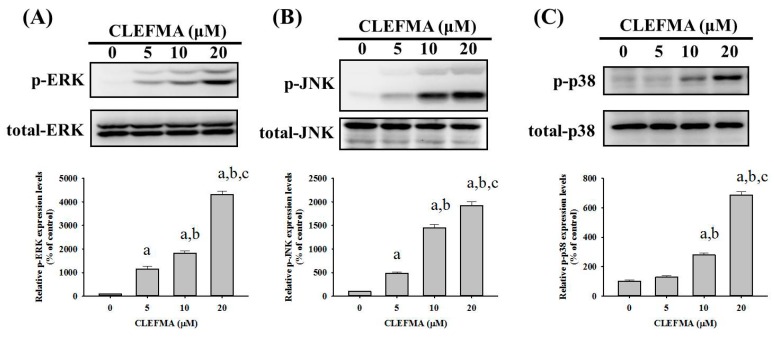
Effects of CLEFMA on the phosphorylation of ERK, c-Jun N-terminal kinases (JNK) and p38 in U2OS cells. Expressions of ERK1/2, JNK 1/2 and p38, as well as their phosphorylation after various concentrations (5, 10 and 20 μM) of CLEFMA treatment for 24 h in U2OS cells, were measured through Western blot analysis. Next, they were subjected to quantitative analysis. Results are shown as mean ± S.D.; *n* = 3. ANOVA analysis with Turkey’s posteriori comparison was used. (**A**) *p*-ERK: F = 275.513, *p* < 0.001; (**B**) p-JNK: F = 205.474, *p* < 0.001; and (**C**) p = p38: F = 292.128, *p* < 0.001. a: Significantly different, *p* < 0.05, when compared to control. B: Significantly different, *p* < 0.05, when compared to 5 μM. c: Significantly different, *p* < 0.05, when compared to 10 μM.

**Figure 6:**
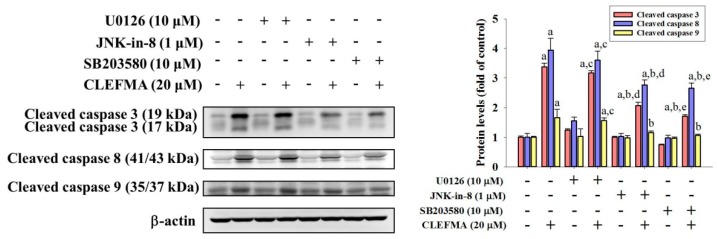
Effects of CLEFMA and inhibitors of ERK1/2 (U0126), JNK1/2 (JNK-in-8), and p38 (SB203580) on cleaved caspases 3, 8 and 9 expression of U2OS cells. Expressions of cleaved caspases 3, 8 and 9 after pretreatment with or without 10 μM of U0126, 1 μM of JNK-in-8, and 10 μM of SB203580 for 1 h followed by 20 μM or without CLEFMA treatment for an additional 24 h in U2OS cells were measured through Western blot analysis. Next, they were subjected to quantitative analysis. Results are shown as mean ± S.D.; *n* = 3. ANOVA analysis with Turkey’s posteriori comparison was used. Cleaved caspase 3: F = 502.398, *p* < 0.001; Cleaved caspase 8: F = 95.967, *p* < 0.001; and cleaved caspase 9: F = 10.543, *p* < 0.001. a: Significantly different, *p* < 0.05, when compared to control. b: Significantly different, *p* < 0.05, when compared to 20 μM CLEFMA. c: Significantly different, *p* < 0.05, when compared to U0126. d: Significantly different, *p* < 0.05, when compared to JNK-in-8. e: Significantly different, *p* < 0.05, when compared to SB203580.

Since MAPK pathways have been implicated as playing an important role in the action of chemotherapeutic drugs in the regulation of apoptosis and may be part of the signaling pathways that directly affect caspases 3, 8, and 9, the Western blot analysis was employed to further investigate the underlying molecular mechanisms. As shown in Figure 5A–C, CLEFMA increased the phosphorylation of ERK1/2, JNK1/2 and p38, dose-dependently, in U2OS cells (*p* < 0.001, *p* < 0.001 and *p* < 0.001, respectively), indicating that CLEFMA activates the phosphorylation of ERK1/2, JNK1/2 and p38 in U2OS cells. Furthermore, to identify whether the activation of ERK1/2, JNK1/2 and p38 phosphorylation by CLEFMA interferes with the actions of caspases 3, 8, and 9 of the extrinsic and intrinsic apoptotic processes in U2OS cells, we used inhibitors of ERK1/2 (U0126), JNK1/2 (JNK-in-8), and p38 (SB203580) with or without treatment with 20 μM CLEFMA in Western blotting. Cleaved caspases 3, 8, and 9 were activated by 20 μM of CLEFMA (*p* < 0.001, *p* < 0.001 and *p* = 0.001), as expected. (Figure 6) Intriguingly, inhibitors of JNK1/2 (JNK-in-8) and p38 (SB203580) significantly repressed CLEFMA’s increase of cleaved caspases 3, 8 and 9 in U2OS cells (JNK-in-8: *p* < 0.001, *p* < 0.001 and *p* = 0.013; SB203580: *p* < 0.001, *p* < 0.001 and *p* = 0.003), but the inhibitor of ERK1/2 (U0126) did not suppress CLEFMA’s increase of cleaved caspases 3, 8 and 9 (U0126: *p* = 0.088, *p* = 0.568 and *p* = 0.990). Overall, these findings indicated that JNK1/2 and p38 pathways play a critical upstream role in CLEFMA-mediated apoptosis of extrinsic caspase 8- and intrinsic caspase 9-mediated pathways and their downstream effector caspase 3 in U2OS cells.

## 3. Discussion

In previous studies, curcumin has been reported to induce the apoptosis of human leukemia THP-1 cells through the activation of JNK/ERK pathways [21] and SHI-1 cells, possibly via both intrinsic and extrinsic pathways triggered by MAPKs (ERK, JNK and p38) signaling [22]. Also, curcumin exerts antitumor effects in retinoblastoma cells by regulating the JNK and p38 pathways [23], while this occurs through ERK1/2 and p38 signaling in malignant mesothelioma cells [24]. In human osteoclastoma cells, curcumin inhibits cell proliferation and promotes apoptosis through JNK, NF-κB and MMP-9 signaling pathways [25]. In spite of its efficacy and safety, curcumin has severely limited bioavailability because of its poor absorption and rapid metabolism [16].

After using the adjuvant to improve the poor bioavailability of curcumin, natural borneol and curcumin synergistically induce the apoptosis of human melanoma A375 cells with the involvement of the downregulation of Akt and ERK1/2 phosphorylation and the upregulation of phosphorylated JNK [26]. Similarly, the JNK/Bcl-2/Beclin1 pathway is thought to play a key role in the induction of apoptosis and autophagic cell death in breast cancer cells by the co-treatment of curcumin and berberine [27]. Additionally, synergistic inhibitory effects of cetuximab and curcumin on human cisplatin-resistant oral cancer CAR cells have been observed through the MAPK pathway and the intrinsic apoptotic process [28]. Moreover, curcumin-based photodynamic therapy induces breast cancer apoptosis through the activation of the ROS-mediated JNK/caspase-3 signaling pathway [29].

In managing patients diagnosed with any form of osteosarcoma, powerful chemotherapeutic drugs are the mainstay. Apart from adjuvants, structural analogues of curcumin (e.g., EF-24 and CLEFMA) have been undertaken to improve the bioavailability of curcumin for chemotherapy [16]. Although the synthetic curcuminoid CLEFMA developed over the past years has focused on anticancer effects against lung cancer cells [17,18,20], no research has been reported on the apoptotic process of CLEFMA in osteosarcoma cells. Here, we intriguingly found that CLEFMA decreases cell viabilities and induces cell apoptosis in human osteosarcoma U2OS and HOS cells.

Currently, the process of apoptosis is triggered by two different signaling pathways. The extrinsic apoptotic signal, which responded mainly to extracellular stimuli, involves death receptors, and the intrinsic apoptotic process, activated by modulators within the cell itself, involves the mitochondria [30,31]. The action of the cascade of caspases is required to conduct apoptosis signal transduction and execution. As in other reports, we discovered that effector caspase 3 plays a critical role in the underlying programs of apoptosis and relies on the activation of its upstream initiators including extrinsic caspase 8 and intrinsic caspase 9 [8,32].

By collecting information from various aspects of signal transduction cascades and cellular metabolism, both pathways continuously process this signaling, and eventually decide on the fate of cells. While CLEFMA’s phosphorylation of ERK1/2, JNK1/2 and p38 in U2OS cells was observed in the study, we supposed that CLEFMA’s induction of the extrinsic and intrinsic apoptotic pathways was achieved through these three MAPK pathways. Unexpectedly, CLEFMA’s increases of cleaved caspases 3, 8, and 9 could be effectively inhibited by co-treatment with inhibitors of JNK (JNK-in-8) and p38 (SB203580), but co-treatment with the ERK inhibitor (U0126) had no effect on the increased effect. Therefore, these findings suggested that CLEFMA activates both extrinsic and intrinsic apoptotic pathways in U2OS cells through JNK and p38 signaling, but the ERK pathway is not involved. CLEFMA’s increases of cleaved caspases 3, 8, and 9 could be effectively inhibited with the co-treatment of the ERK inhibitor (U0126), implying that the cleaved caspases 3, 8, and 9 are not the downstream of the CLEFMA’s phosphorylation of ERK1/2.

## 4. Materials and Methods

### 4.1. Materials

Cell culture materials including Dulbecco’s modified Eagle medium (DMEM) and fetal bovine serum (FBS) were purchased from Gibco-BRL (Gaithersburg, MD, USA) and Hyclone Laboratories, Inc. (Logan, UT, USA), respectively. Antibodies specific for p38, phosphorylated p38, β-actin, caspases 3 and 8, and FITC (fluorescein isothiocyanate-labeled) Annexin V Apoptosis Detection Kit I were obtained from BD Biosciences (San Jose, CA, USA). Human Apoptosis Array Kit was purchased from R&D Systems (Minneapolis, MN, USA). Additionally, antibodies specific for ERK1/2, JNK1/2, phosphorylated ERK1/2 and JNK1/2, caspases 9, and cleaved caspases 3, 8 and 9 were purchased from Cell Signaling Technology (Danvers, MA, USA). Unless otherwise specified, all chemicals used in this study were purchased from Sigma-Aldrich (St. Louis, MO, USA).

### 4.2. Cell Culture and CLEFMA Treatment

Obtained from the Food Industry Research and Development Institute (Hsinchu, Taiwan), the human osteosarcoma U2OS (15-year-old female) cells and HOS (13 year-old female) cells were supplemented with 10% FBS, 1% penicillin/streptomycin, and 5 mL glutamine while being cultured in DMEM and Eagle’s MEM, respectively. The cell cultures were maintained at 37 °C in a humidified atmosphere of a 5% CO2 incubator. CLEFMA was purchased from Sigma-Aldrich (St. Louis, MO, USA).

### 4.3. Microculture Tetrazolium Colorimetric (MTT) Assay

To obtain information regarding the effect of apoptosis induced by CLEFMA, we subjected 8.5 × 10^4^/well U2OS cells and 7.5 × 10^4^/well HOS cells in 24-well plates for 16 h and treated them with different concentrations (5, 10, 20, 40 and 80 μM) of CLEFMA to assay cell viability via MTT [3-(4,5-dimethylthiazol-2-yl)-2,5-diphenyltetrazolium bromide] assay. After the 24 h exposure period, the media were removed and the U2OS and HOS cells were washed with phosphate-buffered saline. Afterwards, the medium was changed and the cells were incubated with MTT (0.5 mg/mL) for 4 h [33,34].

### 4.4. Annexin V-FITC Apoptosis Staining Assay

About 8.5 × 10^5^ U2OS and HOS cells in one 6 cm plate were cultured and treated with different concentrations (0, 5, 10 and 20 μM) of CLEFMA for 24 h. Subsequently, U2OS cells were harvested with trypsinization together with floating non-viable cells. The FITC Annexin V Apoptosis Detection Kit I was used according to the manufacturer’s protocols (BD Biosciences, San Jose, CA, USA); thereafter, the cell cycle analysis was measured by flow cytometry. Combined with PI staining, annexin V-FITC apoptosis staining was performed to differentiate apoptosis from necrosis.

### 4.5. Human Apoptosis Array

To explore the underlying mechanism of induced apoptosis, a Human Apoptosis Array Kit was used to evaluate protein lysates from vehicle- or 20 μM CLEFMA-treated cells for 24 h according to the manufacturer’s protocols (R&D Systems, Minneapolis, MN). The kit detected 35 human apoptosis-related proteins simultaneously. Captured proteins were presented on the nitrocellulose membrane, detected with biotinylated detection antibodies, then finally visualized using chemiluminescent detection reagents.

### 4.6. Protein Extraction and Western Blot Analysis

To investigate the molecular mechanism further, the initiator and effector caspases and signaling pathways were detected using Western blot analysis. We plated 8.5 × 10^5^ U2OS cells in 6 cm plates for 16 h and treated them with different concentrations (0, 5, 10 and 20 μM) of CLEFMA for 24 h, and the total cell lysates of U2OS cells were prepared as described previously [33,34,35]. Western blot analysis was performed using specific primary antibodies against caspases 3, 8 and 9, cleaved caspases 3, 8 and 9, and the specific antibodies for unphosphorylated or phosphorylated forms of the three corresponding MAPKs (ERK1/2, JNK1/2, and p38). As described previously, blots were then incubated with a horseradish peroxidase goat anti-rabbit or anti-mouse IgG for 1 h, and the intensity of each band was measured via densitometry [33,34,35].

### 4.7. Statistical Analysis

Statistical calculations of the data were performed using one-way analysis of variance (ANOVA) with post hoc Scheffe’s and Turkey’s tests for more than two groups with unequal and equal sample sizes per group, respectively. Each experiment was performed in triplicate, and three independent experiments were performed. Statistical significance was at *p* < 0.05.

## 5. Conclusions

Overall, these results demonstrated that CLEFMA decreases cell viabilities and induces the apoptosis of human osteosarcoma U2OS and HOS cells. By activating JNK and p38 pathways, but not via the ERK, both the extrinsic and intrinsic caspase cascades are triggered to induce the apoptosis of U2OS cells. Thus, CLEFMA may be a potential therapeutic agent against human osteosarcoma, whereas the therapeutic potential of CLEFMA combined with chemotherapy in osteosarcoma treatment should warrant evaluation in future research. Further tests are needed to investigate the detailed effects and possible mechanism of CLEFMA on the cell cycle progression and regulatory molecules of human osteosarcoma cells; however, animal studies are needed to justify CLEFMA as a promising candidate as a cytotoxic agent against osteosarcoma in vivo.
